# Polymyxin B-induced gastric spasm:a case report

**DOI:** 10.3389/fped.2026.1839525

**Published:** 2026-06-22

**Authors:** Chuan Sun, Yao Sun, Dandan Yang, Tianjiao Hu, Lihua Yuan

**Affiliations:** Department of Pharmacy, Children’s Hospital of Nanjing Medical University, Nanjing, Jiangsu, China

**Keywords:** adverse reactions, gastric spasm, pediatrics, polymyxin B, wound infection

## Abstract

**Background:**

Polymyxin B is commonly used to treat carbapenem-resistant Acinetobacter baumannii (CRAB) infections, with increasing attention being paid to its effectiveness and adverse reactions. This article reports a rare case of gastric spasm as an adverse drug reaction caused by polymyxin B used for anti-infective treatment.

**Case presentation:**

A 14-year-old boy was diagnosed with a CRAB infection following surgery. He was treated with a combination of meropenem and polymyxin B for the infection, which led to severe abdominal colic symptoms in the patient. A comprehensive investigation into the potential causes of the severe abdominal colic was conducted clinically. Following the discontinuation of polymyxin B, the patient's severe abdominal colic symptoms gradually alleviated. Therefore, polymyxin B was identified as the causative factor for the gastric spasm in this patient.

**Results:**

Polymyxin B may induce gastric spasm in children by elevating histamine levels in the body. This increase in histamine enhances gastric acid secretion and triggers an inflammatory response, which collectively compromises the gastric mucosal barrier and promotes excessive contraction of the gastric smooth muscle.

## Introduction

## Background

Surgical site infections (SSIs) represent the most prevalent complication arising from millions of surgical procedures conducted each year globally (1). SSIs pose a significant challenge within the surgical field, as they are linked to increased mortality, morbidity, prolonged hospitalization, and elevated healthcare costs. Furthermore, SSIs are among the leading causes of patient readmission, posing a serious threat to patient safety and diminishing quality of life during the postoperative period (2). Therefore, early diagnosis and treatment of SSIs are imperative.

Acinetobacter baumannii (AB) is commonly found in hospital environments, on human skin, and in the respiratory tract, and is recognized as a significant pathogen in incisional infections (3). Polymyxin B is frequently employed to treat AB infections that are resistant to conventional antibiotics, including carbapenems, particularly severe infections (4–6). Nevertheless, it is essential to develop individualized treatment regimens that account for patient-specific conditions while closely monitoring for adverse drug reactions (ADRs). Common ADRs to polymyxin B include nephrotoxicity, neurotoxicity, and hyperpigmentation, along with other effects such as blood eosinophilia, severe pain from intramuscular injections, drug fever, urticaria, phlebitis, and diarrhea (7). To our knowledge, there are no existing reports linking polymyxin to gastric spasm.

Here we report a rare case of gastric spasm induced by polymyxin B during anti-infective therapy.

## Case presentation

The 14-year-old male patient, standing at 175 cm and weighing 113 kg, was hospitalized on February 9, 2025, due to trauma resulting in swelling, pain, deformity, and restricted mobility in the right hip joint for over 6 h. He underwent a procedure involving internal fixation and external fixation for a right femoral head fracture under general anesthesia. Following discharge with postoperative improvement, he was readmitted on February 21 due to a split in the right lower limb post-surgery. Examination revealed skin fissures at the surgical site with slight discharge, accompanied by redness, swelling, and tenderness in the surrounding area. Throughout the illness, the patient did not experience fever, vomiting, appetite or sleep disturbances, and had normal bowel movements.

**Figure 1:**
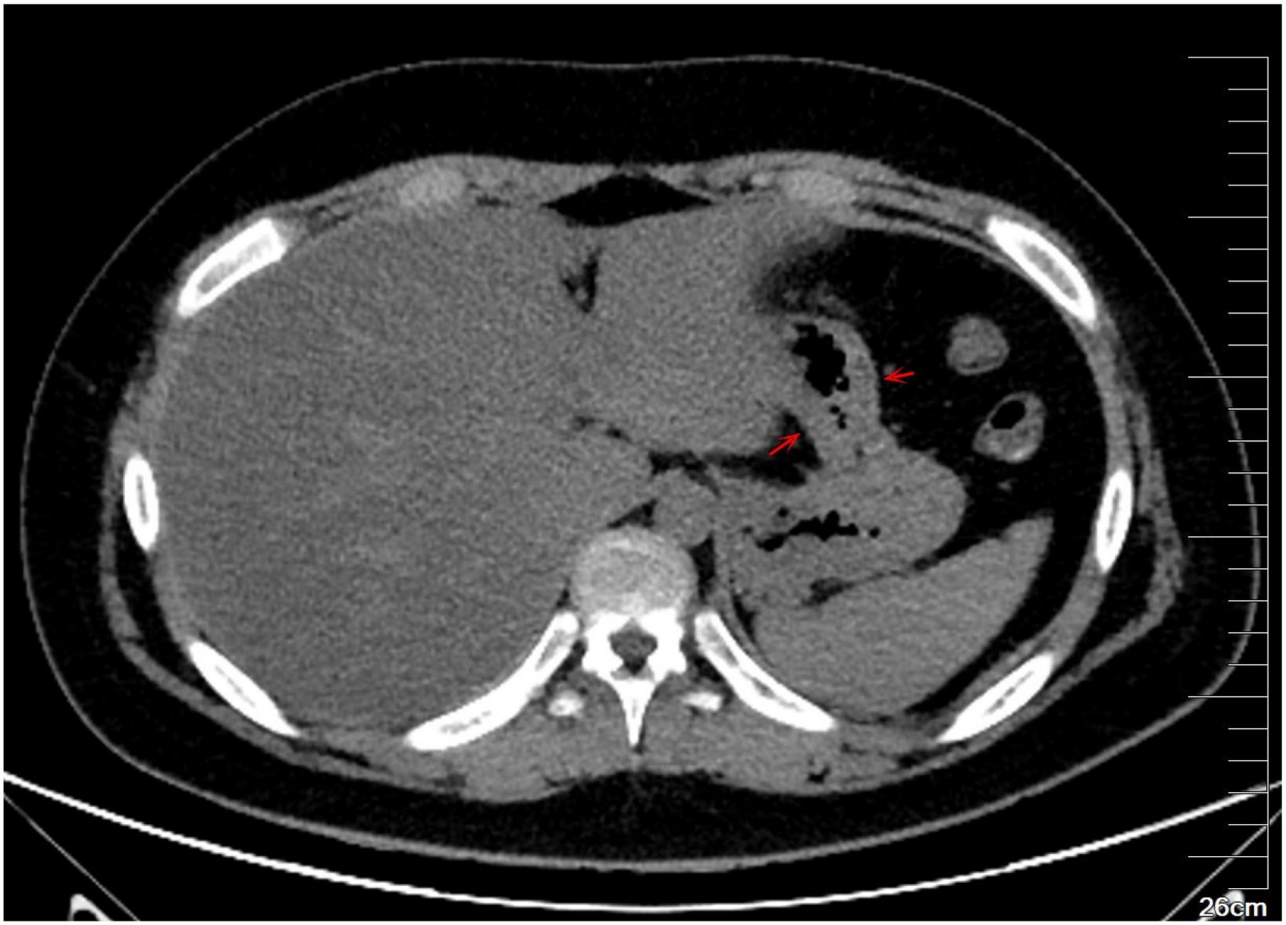
The abdominal CT image shows that the gastric wall has thickened.

On February 23, secretion culture revealed that Pseudomonas aeruginosa demonstrated intermediate susceptibility to ticarcillin-clavulanic acid and sensitivity to the other antibiotics tested, while Acinetobacter baumannii was susceptible only to minocycline, tigecycline, and colistin, and resistant to all remaining agents. Clinically, the patient received meropenem 2 g intravenously every 8 h in combination with polymyxin B 100 mg intravenously every 12 h. The child experienced nausea during the initial polymyxin B infusion. Subsequently, during the second infusion, the child exhibited symptoms including mouth numbness, vomiting, irritability, abdominal hugging, curling up and crying, visible cold sweat on the forehead, severe gastric cramps, and intolerable pain. A computed tomography (CT) of the abdomen showed gastric wall thickening (Figure 1). The patient's parents stated that the patient had no history of stomach diseases. A pain score of 10 on the FLACC (Face, Legs, Activity, Cry, Consolability) pain scale was assessed by the clinical pharmacist at the bedside (Table 1). Discontinuation of polymyxin B was advised, leading to a significant improvement in symptoms upon cessation of the infusion. On February 25, the clinical pharmacist recommended switching the anti-infection regimen to meropenem 2 g administered intravenously over 3 h every 8 h, along with an initial oral dose of minocycline of 0.2 g, followed by 0.1 g every 12 h. Secretion cultures on March 3 and March 6 showed no bacterial growth. The child was discharged on March 9 with satisfactory incision healing, and the medication was adjusted for outpatient management.

**Figure 2:**
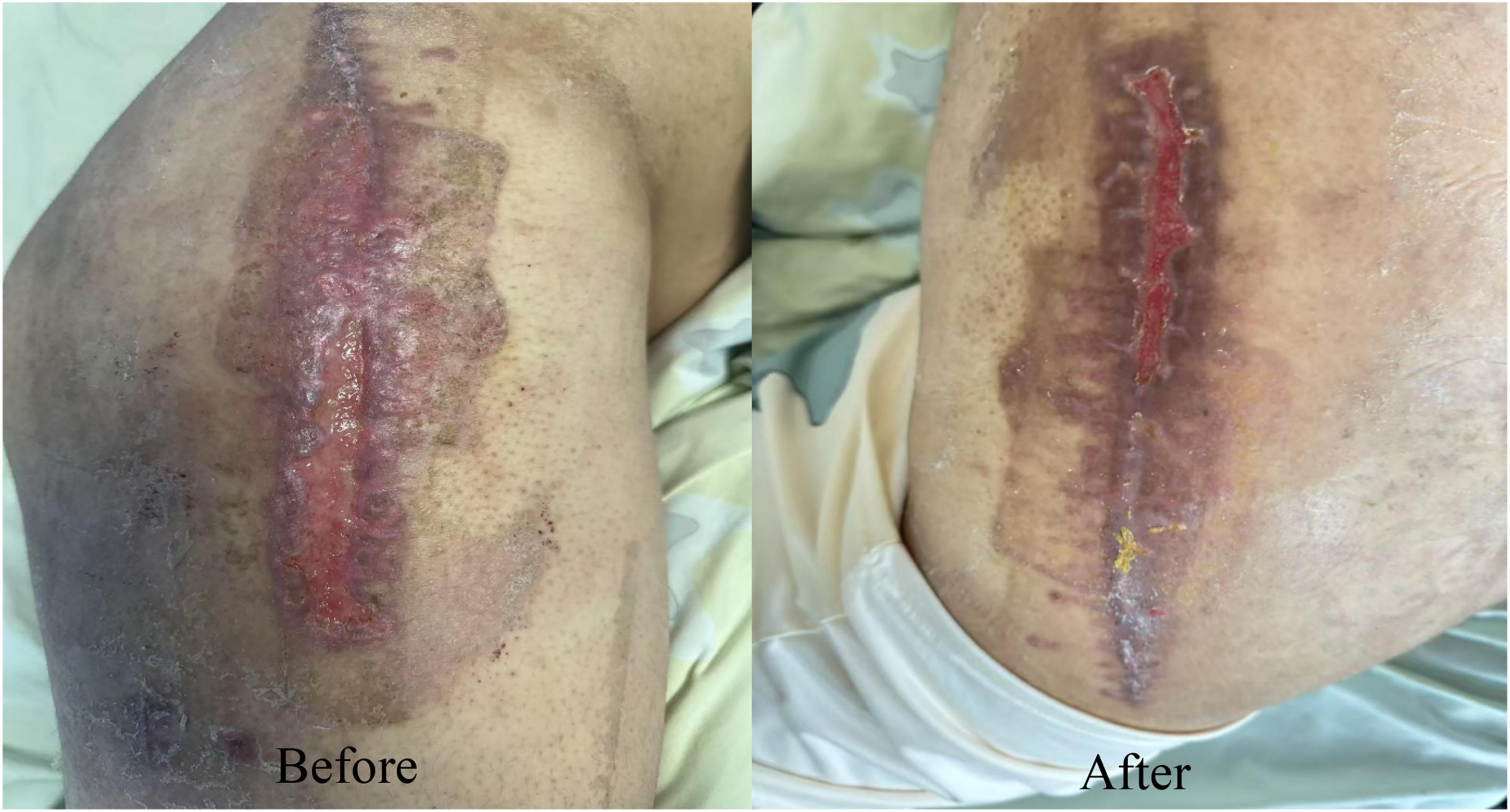
The changes in incision healing in children before and after anti-infection treatment.

**Table 1:** FLACC scale.

Categories	Grand			Scoring
	0	1	2	
Face	No particular expression or smile	Occasional grimace or frown, withdrawn, disinterested	Frequent to constant quivering chin, clenched jaw	2
Legs	Normal position or relaxed	Uneasy, restless, tense	Kicking or legs drawn up	2
Activity	Lying quietly, normal position, moves easily	Squirming, shifting back and forth, tense	Arched, rigid, or jerking	2
Cry	No cry (awake or asleep)	Moans or whimpers, occasional complaint	Crying steadily, screams, or sobs, frequent complaints	2
Consolability	Content, relaxed	Reassured by occasional touching, hugging, or being talked to, distractible	Difficult to console or comfort	2
Total				10

## Discussion and conclusions

Consulting various domestic and international drug manuals and literature on polymyxin B, the most common ADRs include nephrotoxicity, neurotoxicity, and hypersensitivity reactions (8). Initially, its use may lead to ADRs such as nausea, vomiting, and abnormal oral sensations, but reports of gastric spasm are lacking.

In this case, the infected incision in the child significantly improved after treatment (Figure 2). However, during the combined treatment of polymyxin B and meropenem, the child experienced symptoms such as nausea, vomiting, oral numbness, and epigastric pain during two polymyxin B infusions, which improved upon cessation of the infusion. Despite receiving meropenem concurrently, the child only exhibited symptoms upon polymyxin B infusion, which promptly resolved upon discontinuation of the drug. Conversely, no such reaction occurred during meropenem infusion, thus excluding meropenem as the culprit. The child's gastric spasm showed a strong temporal association with polymyxin B, and the CT scan of the abdomen indicated gastric wall thickening, with no gastrointestinal symptoms before treatment. Based on this, we conclude that polymyxin B may induce symptoms of gastric spasm.

The study utilized the Naranjo algorithm to establish the causal link between polymyxin B and gastric spasm, yielding a score of +6, probably implicating polymyxin B in the onset of gastric spasm (Table 2). This finding corroborates the clinical pharmacist's initial assessment. The precise mechanism by which polymyxin B induces gastric spasm remains unclear; however, existing research indicates that polymyxin B functions as a histamine-releasing agent (9). Histamine and its receptors (H1R–H4R) play a crucial and significant role in the development of various allergic diseases. Histamine is produced and discharged by basophils, mast cells, and neurons, with polymyxin B elevating endogenous histamine levels and activating H1R–H4R (10). Activation of the H1R triggers smooth muscle myosin light chain kinase activation, sustaining heightened myosin light chain 20 phosphorylation levels, enhancing calcium sensitization, and facilitating muscle contraction (11). Furthermore, activation of H1R exerts pro-inflammatory effects. Activation of H2R stimulates adenylate cyclase, leading to an increase in intracellular cyclic adenosine monophosphate (cAMP) levels. cAMP, as a second messenger, further activates the proton pump (H⁺-K⁺-ATPase), promoting gastric acid secretion (12). Activation of H4R significantly enhances inflammatory responses in experimental colitis, radiation colitis, intestinal ischemia/reperfusion injury, and allergic reactions. The involvement of H4R activation has been identified in the pathogenesis of peptic ulcers and carcinogenesis (13). Activation of both H4R and H3R enhances the effects of acetylcholine on intestinal motility (14). H4R-mediated mast cell activation leads to the expression of pro-inflammatory cytokines and chemokines such as IL-6, TNF-α, TGF-*β*1, RANTES, IL-8, MIP-1*α*, and MCP-1 (15). We guess that the mechanism underlying the ADR of gastric spasm is attributed to polymyxin B, which elevates histamine levels in the body. This increase in histamine stimulates gastric acid secretion. It triggers an inflammatory response, leading to a dual effect that compromises the gastric mucosal barrier and induces excessive contraction of the gastric smooth muscle, ultimately resulting in gastric spasm in affected children.

**Table 2:** Naranjo's scale for the likelihood of gastric spasm caused by polymyxin B.

Naranjo's scale Question	Yes	No	Don't know	Score for gastric spasm
1. Are there previous conclusive reports on this reaction?	+1	0	0	0
2. Did the adverse event appear after the suspect drug was administered?	+2	−1	0	+2
3. Did the adverse reaction improve when the drug was discontinued or a specific antagonist was administered?	+1	0	0	+1
4. Did the adverse reaction reappear when the drug was re-administered?	+2	−1	0	0
5. Are there alternate causes (other than the drug) that could have solely caused the reaction?	−1	+2	0	+2
6. Did the reaction reappear when a placebo was given?	−1	+1	0	0
7. Was the drug detected in the blood (or other fluids) in a concentration known to be toxic?	+1	0	0	0
8. Was the reaction more severe when the dose was increased or less severe when the dose was decreased?	+1	0	0	0
9. Did the patient have a similar reaction to the same or similar drugs in any previous exposure?	+1	0	0	0
10. Was the adverse event confirmed by objective evidence?	+1	0	0	+1
Total				+6

Scoring for Naranjo's algorithm; ≥9 = definite; 5–8 = probable; 1–4 = possible; 0 = doubtful.

A notable limitation of this case is the absence of a drug re-excitation test. A positive result from such a test would have strengthened the association between polymyxin B and gastric spasm. However, considering the child's distressing gastric spasm and the availability of alternative antibiotics, we opted not to reintroduce polymyxin B due to ethical and patient safety concerns. Consequently, this report could not achieve a higher level of causal evidence. We acknowledge that this limitation confines the classification of the adverse reaction to the “ probable ” level; nevertheless, the finding holds significant pharmacovigilance value. Reports of gastric spasm associated with polymyxin B are scarce in the literature, and this case serves to alert clinicians to this potential adverse effect. 

As the use of polymyxin B has increased, several previously rare and undetected ADRs have emerged. Future research should aim to further quantify the incidence of these adverse events and validate our newly discovered conclusion that polymyxin B can induce gastric spasm.

## Data Availability

The original contributions presented in the study are included in the article/Supplementary Material, further inquiries can be directed to the corresponding author.
