# Correction: Polymyxin B-induced gastric spasm: a case report

**DOI:** 10.3389/fped.2026.1923633

**Published:** 2026-07-29

**Authors:** Chuan Sun, Yao Sun, Dandan Yang, Tianjiao Hu, Lihua Yuan

**Affiliations:** Department of Pharmacy, Children’s Hospital of Nanjing Medical University, Nanjing, Jiangsu, China

**Keywords:** adverse reactions, gastric spasm, pediatrics, polymyxin B, wound infection

**Error in figure/table**

There was a mistake in table 2 as published. There are discrepancies in the scores given for questions 10 and 4, as well as the final total score, in this table. The corrected table 2 appears below.

**Table 2:** Naranjo's scale for the likelihood of gastric spasm caused by polymyxin B.

Naranjo's scale Question	Yes	No	Don't know	Score for gastric spasm
1. Are there previous conclusive reports on this reaction?	+1	0	0	0
2. Did the adverse event appear after the suspect drug was administered?	+2	−1	0	+2
3. Did the adverse reaction improve when the drug was discontinued or a specific antagonist was administered?	+1	0	0	+1
4. Did the adverse reaction reappear when the drug was re-administered?	+2	−1	0	0
5. Are there alternate causes (other than the drug) that could have solely caused the reaction?	−1	+2	0	+2
6. Did the reaction reappear when a placebo was given?	−1	+1	0	0
7. Was the drug detected in the blood (or other fluids) in a concentration known to be toxic?	+1	0	0	0
8. Was the reaction more severe when the dose was increased or less severe when the dose was decreased?	+1	0	0	0
9. Did the patient have a similar reaction to the same or similar drugs in any previous exposure?	+1	0	0	0
10. Was the adverse event confirmed by objective evidence?	+1	0	0	+1
Total				+6

Scoring for Naranjo's algorithm; ≥9 = definite; 5–8 = probable; 1–4 = possible; 0 = doubtful.

The original version of this article has been updated.

